# Cardiovascular and Metabolic Disease: New Treatment and Future Directions—The 3rd Edition

**DOI:** 10.3390/biomedicines13081914

**Published:** 2025-08-06

**Authors:** Alfredo Caturano

**Affiliations:** Department of Human Sciences and Promotion of the Quality of Life, San Raffaele Roma University, 00166 Rome, Italy; alfredo.caturano@uniroma5.it; Tel.: +39-3338616985

## 1. Introduction

Cardiovascular and metabolic diseases remain the most pressing global health concerns of our time, with a profound impact on both individual lives and healthcare systems [1]. According to recent data, cardiovascular diseases (CVDs) were responsible for nearly 18.6 million deaths globally in 2019, accounting for approximately one-third of all fatalities [2]. This alarming burden is closely intertwined with the global rise in metabolic disorders, most notably diabetes, which affects over 420 million individuals and contributes directly to an additional 1.5 million deaths annually [3]. These conditions rarely occur in isolation and often coexist, forming a dangerous clinical synergy that accelerates disease progression and worsens patient outcomes [4]. This bidirectional link between metabolic dysfunction and cardiovascular disease highlights the importance of a comprehensive, integrated approach to prevention and care [5]. Lifestyle-related risk factors, such as tobacco use, poor nutrition, physical inactivity, and excessive alcohol consumption, continue to play a central role in the onset and progression of both disease groups [6]. However, recent years have also brought significant advancements in therapeutic strategies. Notably, the emergence of sodium–glucose cotransporter-2 inhibitors (SGLT2i) and glucagon-like peptide 1 (GLP-1) receptor agonists has revolutionized the management of both diabetes and heart failure, while evolving interventional techniques are reshaping the clinical landscape [7,8,9]. This Special Issue brings together some of the most promising scientific insights in this rapidly advancing field.

## 2. Overview of Published Articles

This Special Issue presents a diverse and methodologically robust collection of 34 scientific articles, including original research, reviews, and experimental studies. addressing the broad and interwoven spectrum of cardiovascular and metabolic diseases. The contributions reflect a multidisciplinary approach that spans from molecular biology to advanced imaging, from novel therapeutic strategies to real-world clinical data. Below, we highlight representative works from this Special Issue, grouped by thematic domain and described individually to reflect their distinct scientific impact.

### 2.1. Biomarkers and Molecular Mechanisms

Alexander E. Berezin et al. (contribution 1) investigated the predictive role of irisin, a myokine involved in energy homeostasis, in patients with heart failure with reduced ejection fraction (HFrEF). In a prospective study of 313 patients, they demonstrated that higher baseline serum irisin levels were independently associated with improved left ventricular ejection fraction at 3-month follow-up, outperforming even NT-proBNP in certain predictive models. This study positions irisin as a potential biomarker for cardiac recovery in HFrEF.

Irene Martínez-García et al. (contribution 2) investigated the role of advanced glycation end products (AGEs) in mediating the association between glycated hemoglobin (HbA1c) and aortic pulse wave velocity (a-PWV), an indicator of arterial stiffness, in a cohort of 390 healthy Spanish adults. Using skin autofluorescence (SAF) to measure AGEs, they found that while HbA1c levels were initially associated with increased a-PWV, this effect was largely mediated by SAF. Mediation analysis showed that SAF accounted for over 40% of the relationship, suggesting that AGE accumulation plays a significant role in vascular changes even in healthy individuals.

Caitlin P.S. Ellis et al. (contribution 3) examined protein signatures in perivascular adipose tissue (PVAT) of patients with and without coronary artery disease. Using proteomics and histomorphometric analyses, they identified distinct protein profiles in PVAT related to metabolic signaling, inflammation, and exosome activity. The work provides new insight into adipose tissue heterogeneity and its role in atherogenesis.

Kareem Awad et al. (contribution 4) explored the effects of hyperglycemia on human macrophages and uncovered that high glucose conditions activate the TGF-β1/Smad1/5 signaling pathway, potentially contributing to atherogenic responses. Their findings underscore the role of glucose-induced immune dysregulation in diabetes-related vascular complications.

Isabella D. Cooper et al. (contribution 5) examined the metabolic impact of temporarily suppressing ketosis in a group of healthy, lean, pre-menopausal women who had maintained long-term nutritional ketosis (NK). In a three-phase study, participants transitioned from NK to a 21-day period of suppressed ketosis (SuK) and then returned to NK. During SuK, significant increases were observed in insulin, glucose, HOMA-IR, leptin, and osteocalcin levels, along with reductions in beta-hydroxybutyrate and GLP-1. Importantly, all biomarkers reverted to baseline upon resuming NK, indicating preserved metabolic flexibility. The findings suggest that sustained NK may offer protective effects against hyperinsulinemia without impairing metabolic adaptability.

Manjula Nandakumar et al. (contribution 6) explored the impact of hypoglycemia and subsequent rebound hyperglycemia on cardiovascular risk biomarkers using proteomic profiling. They compared two controlled studies, one involving mild hypoglycemia and the other severe hypoglycemia followed by glucose-induced rebound; selected protein markers were measured in both type 2 diabetes patients and healthy controls. Severe hypoglycemia triggered transient increases in ANGPT1 and DKK1 in controls and suppression of BOC in diabetic participants. Notably, all alterations resolved within one hour, and rebound hyperglycemia did not induce further changes. These results suggest that acute hypoglycemia, rather than glycemic fluctuations, may be the critical driver of short-term cardiovascular risk.

### 2.2. Cardiometabolic and Endocrine Disorders

Jazmine A. Eccles-Miller et al. (contribution 7) explored the sexually dimorphic role of the enzyme CYP2B6 in fasting-induced hepatic steatosis using humanized transgenic mice. Despite previous links between CYP2B6 and anti-obesity effects, both male and female hCYP2B6-Tg mice developed greater steatosis compared to knockout controls when fasted. While treatment with oxylipins 9-HODE and 9-HOTrE altered serum lipids, especially in females, they did not significantly affect liver fat or gene expression. Females showed greater hepatic lipid retention and broader gene expression changes than males. The study concludes that CYP2B6 contributes to sex-specific susceptibility to steatosis, though oxylipins are not the primary mediators of this effect.

Jan Soukop et al. (contribution 8) evaluated the combined effect of fenofibrate and silymarin in an animal model of hereditary hypertriglyceridemia. While fenofibrate alone improved lipid metabolism, the addition of silymarin significantly enhanced antioxidant activity and reduced lipoperoxidation in liver and heart tissues. This synergistic effect supports the potential of combining nutraceuticals with conventional therapies in metabolic syndrome.

Balázs Sági et al. (contribution 9) assessed the prognostic impact of metabolic syndrome (MetS) and its components on cardiovascular and renal outcomes in patients with IgA nephropathy (IgAN). In a long-term study involving 145 CKD patients followed for over 15 years, those with MetS experienced significantly higher rates of adverse events, including both renal and cardiovascular endpoints. Key predictors of poor outcomes included dyslipidemia, diabetes, low hemoglobin, and elevated albuminuria. The presence of multiple MetS components further amplified risk. These findings highlight the importance of early metabolic risk assessment and management in improving long-term outcomes for patients with IgAN.

Ruike Yan and Yanli Cao (contribution 10) assessed the safety and efficacy of epigallocatechin gallate (EGCG) supplementation in obesity and non-alcoholic fatty liver disease (NAFLD). The review synthesizes data from human trials, pointing to dose- and time-dependent benefits on metabolic parameters, with a cautionary note regarding hepatotoxicity at higher concentrations.

Ferenc Sztanek et al. (contribution 11) provided a comprehensive review of emerging pharmacological strategies for the treatment of obesity and type 2 diabetes mellitus (T2DM), focusing on the evolving role of incretin-based therapies. While GLP-1 receptor agonists have shown clinical success, their efficacy may be limited by receptor desensitization. The authors highlight novel developments, including oral GLP-1 agonists and next-generation dual and triple receptor agonists, such as GLP-1/GIP and GLP-1/GIP/glucagon combinations, which promise enhanced metabolic effects. By examining cellular signaling and inflammation pathways, the review underscores the expanding therapeutic landscape and the future potential of precision pharmacotherapy in managing obesity and T2DM.

Salvatore Nesci and Speranza Rubattu (contribution 12) presented an in-depth overview of the multifaceted roles of mitochondrial uncoupling protein 2 (UCP2), highlighting its dual physiological and pathological effects. Unlike UCP1, UCP2 does not mediate traditional proton leakage but appears to modulate oxidative phosphorylation through mild uncoupling and the exchange of C4 metabolites. This dual function allows UCP2 to reduce oxidative stress and potentially support healthy aging and cardiovascular protection. However, its activity may also impair mitochondrial ATP production and insulin secretion. The review underscores UCP2’s complex and context-dependent bioenergetic impact, which remains incompletely understood.

### 2.3. Diagnostic Imaging and Interventional Techniques

Piotr Wańczura et al. (contribution 13) proposed a non-destructive method for visualizing atherosclerotic plaques using MRI paired with an iodine-based contrast agent. Their approach allows detailed, volumetric analysis while preserving tissue integrity, offering a promising complement to standard histology in assessing atherosclerotic burden.

Narae Kim et al. (contribution 14) examined the prognostic utility of carotid intima-media thickness and coronary artery calcium scores in patients with negative treadmill stress echocardiography. Both imaging markers independently predicted adverse cardiovascular outcomes, reinforcing their value in refining risk assessment.

Jaroslav Chlupac et al. (contribution 15) tested the FRAME mesh as external support for vein grafts in a porcine carotid model. The mesh significantly improved graft patency and reduced midgraft neo-intimal hyperplasia compared to controls, suggesting a protective role against stenosis in bypass surgery.

Natalia Zdebik et al. (contribution 16) demonstrated that T1-mapping cardiac MRI sequences can reveal elevated extracellular volume (ECV) in patients with hypertrophic cardiomyopathy (HCM) even in the absence of visible injury on late gadolinium enhancement. Their findings suggest T1-mapping offers valuable diagnostic insights into myocardial changes undetectable by conventional imaging.

Robert J. Gil et al. (contribution 17) reported four-year results from the POLBOS 3 trial comparing the BiOSS LIM C dedicated bifurcation stent to standard drug-eluting stents in non-left main coronary bifurcations. While no significant differences in major adverse cardiovascular events were observed, the BiOSS stent showed high implantation success and comparable long-term safety, establishing it as a viable option for complex bifurcation lesions.

### 2.4. Epidemiology, Population Studies, and Public Health

Vaida Šileikienė et al. (contribution 18) analyzed cardiovascular risk trends in over 52,000 middle-aged Lithuanian men from 2009 to 2019. Despite a decline in hypertension prevalence, nearly half had the condition, with many unaware or untreated. Men with resistant hypertension showed the highest burden of risk factors, particularly obesity, inactivity, and dyslipidemia, underscoring the need for targeted prevention and early intervention strategies.

Elke Boxhammer et al. (contribution 19) explored the impact of prediabetes on survival after ST-elevation myocardial infarction. In a cohort of 725 patients, those with prediabetes had significantly worse three-year survival compared to non-diabetics, with outcomes nearing those of diabetic patients. The findings highlight the need for early recognition and aggressive cardiovascular risk management in individuals with prediabetes.

Mara Volpentesta et al. (contribution 20) assessed the impact of one-year CPAP therapy in elderly patients with obstructive sleep apnea syndrome (OSAS). In a real-life cohort of 469 individuals, CPAP significantly improved respiratory indices, metabolic markers, and vascular function, particularly arterial stiffness and endothelial health. These results support CPAP as a key intervention to mitigate cardiovascular risk in elderly OSAS patients.

Ramcharan Singh Angom et al. (contribution 21) reviewed the advantages of zebrafish as a model for cardiovascular and metabolic diseases. Thanks to their genetic similarity to humans, transparency, and compatibility with gene editing, zebrafish are ideal for studying disease mechanisms, drug screening, and precision medicine. The model’s scalability and cost-effectiveness make it a powerful tool for future translational research.

### 2.5. Translational and Experimental Medicine

Yuxin Nie et al. (contribution 22) explored the mechanisms behind hemodialysis-induced myocardial stunning (HIMS) using a rat model of chronic kidney disease. The study demonstrated that mitochondrial dysfunction and ion imbalances, particularly in potassium and calcium, contribute to HIMS. Activation of mitochondrial ATP-sensitive potassium channels with diazoxide improved both mitochondrial and cardiac function, suggesting a protective strategy against HIMS.

Alexandra Ioana Popescu et al. (contribution 23) investigated the role of microRNAs in patients with chronic limb-threatening ischemia (CLTI), a severe stage of peripheral arterial disease. By analyzing the expression of miR-199a, miR-20a, and miR-30c before and after revascularization, the study highlights their potential as biomarkers for disease monitoring and as future therapeutic targets in the management of critical limb ischemia.

Nicholas Pavlatos and Dinesh K. Kalra (contribution 24) reviewed the role of lipoprotein(a) in peripheral artery disease (PAD), highlighting its pro-atherogenic, pro-inflammatory, and pro-thrombotic properties. Despite its strong causal link to cardiovascular diseases, lipoprotein(a) remains underrecognized in PAD. The article emphasizes the need for greater clinical awareness and explores emerging therapies aimed at reducing related vascular risks.

Ekaterina Ogurtsova et al. (contribution 25) reviewed murine models used to study the cardiometabolic phenotype of heart failure with preserved ejection fraction (HFpEF), a condition often linked to obesity, type 2 diabetes, and hypertension. The article explores key pathophysiological mechanisms, such as inflammation, oxidative stress, and metabolic dysfunction, offering insights that may guide future therapeutic strategies for this increasingly prevalent and complex condition.

Diana-Aurora Arnautu et al. (contribution 26) provided a narrative review on inherited dilated cardiomyopathies (DCM), emphasizing the growing role of genetic assessment in risk stratification and treatment personalization. The review discusses the complex genotype–phenotype relationships in DCM and highlights how genetic variants influence disease expression, prognosis, and therapeutic response. Tailoring management based on genetic findings is presented as a key step toward more precise and effective care for affected individuals and their families.

## 3. Therapeutic Innovations and Pharmacological Strategies

Nicia I. Profili et al. (contribution 27) reviewed the emerging role of SGLT2i in patients following acute myocardial infarction. Beyond their glucose-lowering effects, SGLT2i have shown benefits in heart failure management and cardiovascular risk reduction. The article highlights their potential to improve outcomes post-infarction by modulating inflammation, reducing arrhythmias, and supporting ventricular remodeling, especially in patients undergoing percutaneous coronary intervention.

Vasiliki Katsi et al. (contribution 28) reviewed current weight loss therapies and their effects on hypertension. By examining pharmacologic agents, such as GLP-1 receptor agonists, tirzepatide, and combination drugs—as well as bariatric surgery—the article outlines how each intervention influences blood pressure through shared mechanisms such as insulin resistance and neurohormonal dysregulation. The review supports weight loss as a strategic component in hypertension management.

Kyosuke Murai et al. (contribution 29) reviewed clinical evidence on ranolazine, an antianginal agent with notable antiarrhythmic properties. The drug has shown efficacy in reducing ventricular tachycardia episodes and enhancing atrial fibrillation conversion when used alongside agents such as amiodarone. With a favorable safety profile and low proarrhythmic risk, ranolazine emerges as a promising adjunct in arrhythmia management.

Yotam Kolben et al. (contribution 30) assessed the use of wearable technology for real-time hemodynamic monitoring in hemodialysis patients. Their study showed that early and significant drops in blood pressure and other vitals predicted intradialytic hypotension (IDH) episodes. Continuous monitoring proved reliable and may offer a valuable tool for early IDH detection and prevention in clinical practice.

Irina Tarasova et al. (contribution 31) evaluated the impact of multi-task cognitive training (MTT) on brain activity and neurovascular markers in patients after coronary artery bypass surgery. Compared to controls, patients receiving MTT showed improved EEG patterns and favorable shifts in biomarkers such as reduced S100β and increased BDNF. These findings suggest that short-term cognitive training may aid in post-surgical neurocognitive recovery.

Gianfranco Piccirillo et al. (contribution 32) applied artificial intelligence to assess ECG-derived repolarization and non-invasive hemodynamic markers in elderly patients with decompensated chronic heart failure. Higher Tend (Te) intervals were associated with increased mortality, and changes in bioimpedance and repolarization metrics reflected treatment response. These accessible, non-invasive parameters offer promise for remote monitoring and early detection of clinical deterioration using AI-driven tools.

## 4. Systematic Reviews and Meta-Analyses

Amilia Aminuddin et al. (contribution 33) conducted a systematic review and meta-analysis to assess the prognostic value of soluble LOX-1 (sLOX-1) in predicting cardiovascular outcomes. Elevated sLOX-1 levels were associated with an increased risk of major adverse cardiovascular and cerebrovascular events in patients with coronary artery disease. While results support its potential as a biomarker, the authors highlight the need for standardized research to confirm its role in clinical risk stratification.

Bogdan-Flaviu Buz et al. (contribution 34) conducted a systematic review evaluating the effects of hypoglycemic medications on left ventricular remodeling in diabetic patients. The analysis revealed that agents such as liraglutide and dapagliflozin significantly improved cardiac function and reduced ventricular dimensions, while others showed minimal impact. These findings suggest that certain glucose-lowering drugs may offer added cardiovascular benefits beyond glycemic control, supporting a more individualized approach in diabetes management.

## 5. Future Perspectives

Looking ahead, the intersection of cardiovascular and metabolic research is set to benefit from a wave of innovation, fueled by the growth of personalized medicine and precision health [10,11]. Genomic profiling, molecular diagnostics, and individualized risk stratification are opening the door to more targeted and effective interventions, moving beyond a “one-size-fits-all” approach and toward therapies that reflect the unique biology and lifestyle of each patient [12]. The therapeutic frontier is also expanding to include breakthrough technologies such as gene editing, regenerative medicine, and cell-based therapies, offering hope for disease modification and functional restoration in patients with advanced or refractory conditions [13]. At the same time, digital health tools, including wearable sensors, mobile applications, and remote monitoring platforms, are transforming the delivery of care by promoting continuity, accessibility, and patient engagement across geographic and socioeconomic barriers [14,15,16]. Nonetheless, the full realization of these opportunities requires us to confront enduring challenges. Socioeconomic disparities, unequal access to preventive services, and limited health literacy continue to undermine public health efforts [17]. Future research and policy must work in tandem to close these gaps by promoting early lifestyle interventions, investing in community education, and ensuring that innovation reaches all populations equitably [18]. With a multidisciplinary commitment to both scientific rigor and social responsibility, the future of cardiovascular and metabolic care holds the potential for meaningful, lasting impact.

## Data Availability

No dataset was generated for the publication of this article.
